# Advances in cryo-EM that have shaped mechanistic models of membrane-attack-complex assembly and regulation

**DOI:** 10.1107/S2052252526002927

**Published:** 2026-04-23

**Authors:** Dylan P. Noone, Doryen Bubeck

**Affiliations:** a Department of Life Sciences, Sir Ernst Chain Building Imperial College London LondonSW7 2AZ United Kingdom; Boston University School of Medicine, USA

**Keywords:** cryo-EM, complement, membrane attack complexes, CD59

## Abstract

The complement system is a blood-based immune network that plays a crucial role in fighting infection and maintaining immune homeostasis. The membrane attack complex (MAC) is a pore assembled from complement proteins that creates holes in cells when the immune system is activated. Over the last ten years, advances in cryo-electron microscopy (cryo-EM) have enabled key molecular insights into how the MAC assembles, remodels membranes and is regulated. These new tools revealed the inherent flexibility of complement complexes. By adapting computational approaches that disentangle diverse conformations, these studies have provided detailed mechanisms for MAC activity that could underpin novel complement-targeted therapeutics. Now accelerated by AI-driven image analysis and advances in structural cell biology, the next revolution in cryo-EM offers new opportunities to understand the cellular consequences of immune activation.

## Abstract 2

Advances in cryo-electron microscopy have enabled molecular insights into the assembly and regulation of the complement membrane attack complex.

## 1. Introduction

The complement system is a protein-based immune network that plays a pivotal role in human health and disease. Like many biomedically important soluble proteins, X-ray crystallography provided molecular insights that could be pharmacologically exploited. Structures of C3 (Janssen *et al.*, 2005 ▸) and its activated cleavage product, C3b (Janssen *et al.*, 2006 ▸), underpinned the development of compstatin (Lamers *et al.*, 2022 ▸), a cyclic peptide therapeutic used to treat complement disorders. Despite the success of C3 inhibitors, crystallographic approaches to understand molecular interactions of other complement proteins remained limited. Most complement activation happens on cellular surfaces and results in heterogenous and flexible protein assemblies. Structures of these complexes were more suited to investigation by cryo-electron microscopy (cryo-EM), which could visualize membrane proteins in a native environment and did not require crystallization.

Ten years ago, we witnessed a resolution revolution that catapulted cryo-EM from what was colloquially called ‘blobology’ to a technique capable of revealing molecular mechanisms that could inform drug discovery (Kühlbrandt, 2014 ▸). Technological developments in direct electron detection (DED) increased the speed of image acquisition and reduced the noise level in each exposure (Faruqi & Henderson, 2007 ▸). Alongside hardware advances were new software tools that handled noise better and prevented over-refinement of data (Scheres, 2012 ▸), an issue that had been plaguing the community for decades. These advances were particularly impactful for the complement terminal pathway, which culminates in the formation of a large lytic immune pore called the membrane attack complex (MAC). This review highlights how technological advances in cryo-EM have led to breakthroughs in our understanding of MACs and offers a forward-looking perspective on the future of complement cell biology.

## 2. MAC assembly

**Figure 1:**
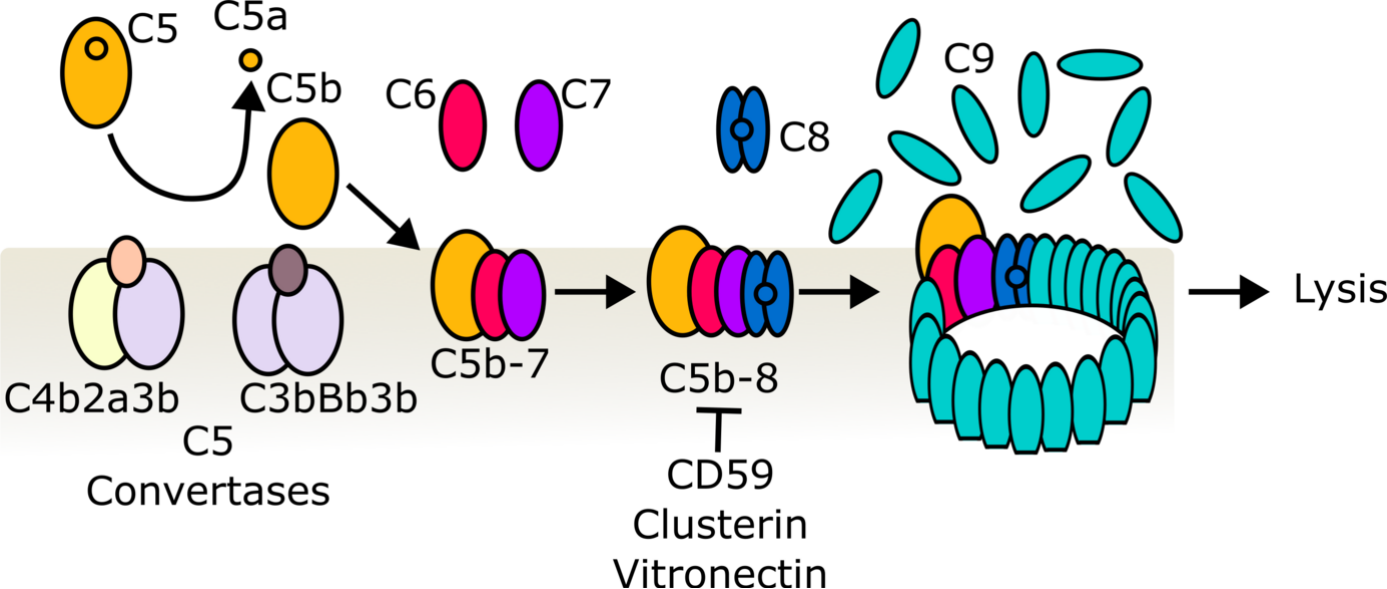
Schematic drawing of MAC assembly. Activation of the alternative pathway or the classical/lectin pathway generates C5 convertases (C3bBb3b and C4b2a3b, respectively) that cleave C5; the resulting C5b (orange) binds C6 (red) at the membrane. C5b6 recruits C7 (purple), C8 (blue) and C9 (turquoise), which polymerizes to form a giant β-barrel pore in lipid bilayers. CD59, clusterin and vitronectin are complement regulators that inhibit MAC formation.

The MAC is the direct killing arm of the complement pathway, creating pores in lipid bilayers of either host or pathogen cells depending on where it is initiated. Recognition of antigens and carbohydrates on the surface of pathogens, or the spontaneous hydrolysis of C3, leads to MAC activation. These three pathways amplify a surface-bound convertase that irreversibly triggers the proteolytic cleavage of complement component 5 (C5) to initiate the MAC (DiScipio *et al.*, 1983 ▸). Cleavage of C5 exposes a transient binding site that can engage other complement proteins including C6. The crystal structure of this initial activation complex, C5b6, showed how domains extending beyond the pore-forming domain of C6 wrap around the thioester warhead of C5 en route towards the target cell (Hadders *et al.*, 2012 ▸). However, it remained unclear how the five complement proteins of the terminal pathway assemble into a membrane-bound complex that ruptures the lipid bilayer (Fig. 1 ▸).

**Figure 2:**
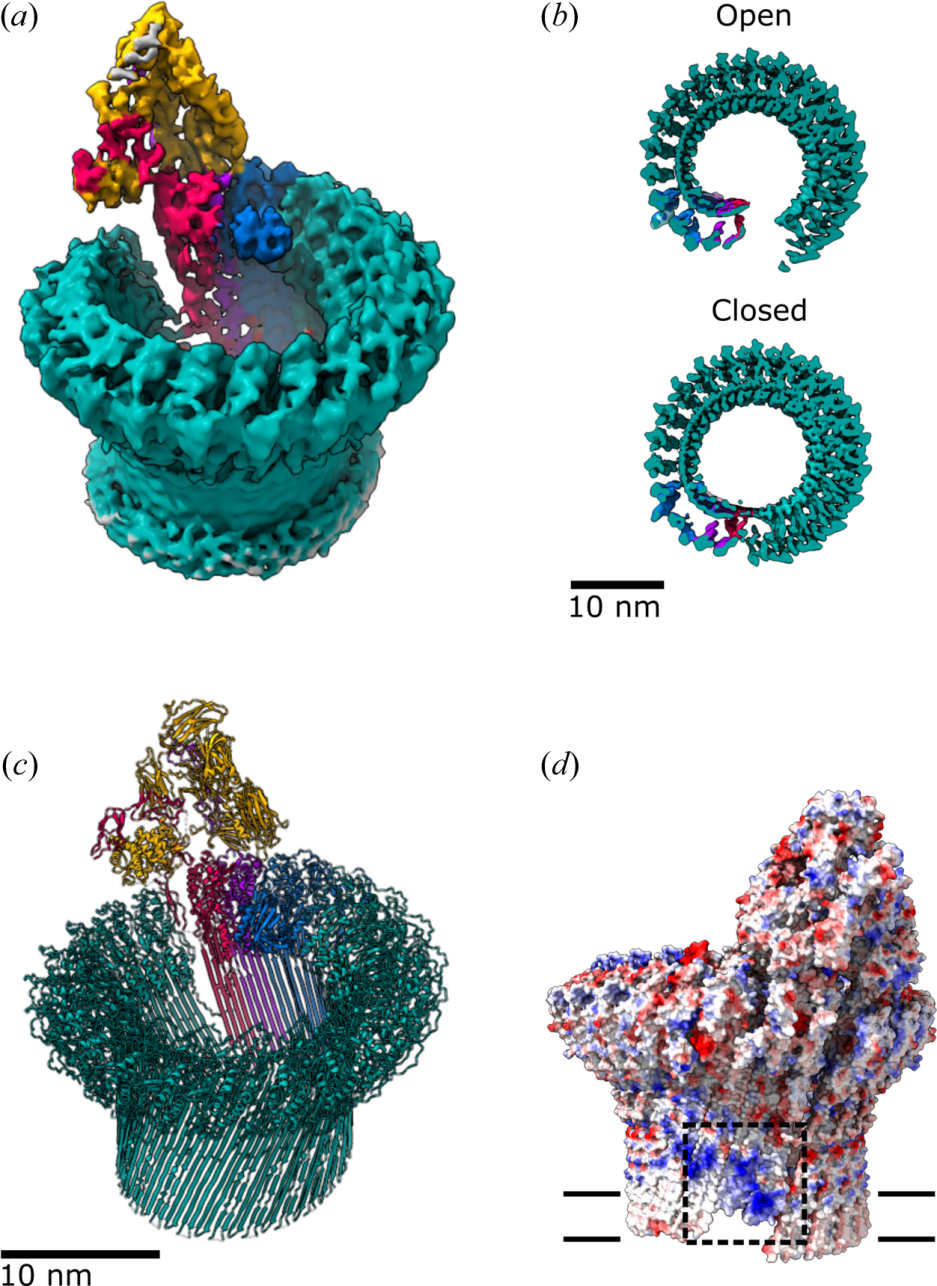
(*a*) First cryo-EM MAC structure (EMDB entry EMD-3134, 8.5 Å; Serna *et al.*, 2016 ▸). (*b*) Cryo-EM structures of open (EMDB entry EMD-0106, 5.6 Å, top; Menny *et al.*, 2018 ▸) and closed conformations (EMDB entry EMD-0107, 5.6 Å, bottom; Menny *et al.*, 2018 ▸) of the MAC pore. (*c*) Atomic model of the MAC closed conformation (PDB entry 6h04; Menny *et al.*, 2018 ▸). Complement proteins coloured as in Fig. 1 ▸. (*d*) Coulombic electrostatic potential ranging from −10 (red) to 10 (blue) kcal (mol·e)^−1^ calculated from the model shown in (*c*). The position of the membrane relative to the models is indicated by double black lines. The boxed region highlights a patch of positively charged residues within the membrane-interacting regions of C6, C7 and C8.

Using cryo-EM to visualize complexes in a membrane environment opened new lines of investigation into MAC assembly. A 23 Å resolution cryo-electron tomography structure of the MAC and assembly intermediates on model membranes highlighted the complexity (Sharp *et al.*, 2016 ▸) and heterogeneity of these systems. In parallel, a single-particle approach of detergent-solubilized pores provided the first insight into MAC stoichiometry and how regulatory domains of complement proteins control the release of pore-forming residues (Serna *et al.*, 2016 ▸) [Fig. 2 ▸(*a*), Supplementary Movie 1]. The cryo-EM structure of the MAC showed that the pore is comprised of a single copy of the activation complex C5b6 together with complement proteins C7 and C8. After localizing to the membrane, 18 copies of C9 polymerize to complete the pore. While these structures answered longstanding questions on MAC composition, the resolution of initial structures remained limited to 8.5 Å despite data being collected with state-of-the-art detectors. Although DEDs substantially improved the signal-to-noise ratio of cryo-EM images, this did not prove to be a silver bullet for solving a high-resolution MAC structure.

As with many dynamic molecular machines, the most interesting parts of the MAC structure were the ones that were not visible in these first maps. Single-particle analysis improves the signal-to-noise ratio of inherently noisy cryo-EM data by combining tens of thousands of identical protein-particle images. However, combining images of flexible assemblies or partially occupied complexes degrades the quality of the reconstruction. As a result, density for flexible regions is weaker and approaches noise levels in the map. More data and better tools for classifying images were needed to achieve a sufficient resolution to understand the molecular drivers underpinning MAC assembly.

Increased data-acquisition speeds and better access to high-end electron microscopes at national facilities enabled the analysis of larger MAC datasets. Aberration-free image shift (AFIS) is an optical mode of data collection that uses beam shifts to acquire a series of exposures after a single stage movement. AFIS led to a step change in imaging speed without compromising quality of the data, alleviating a critical bottleneck in instrument access. By merging several high-quality MAC datasets, there were finally enough data to begin uncovering the dynamic and flexible nature of the MAC pore (Menny *et al.*, 2018 ▸). New advances in Bayesian-based image processing methods allowed 3D classification of these images (Scheres, 2016 ▸). As a result, two distinct groups of pores emerged differing in their extent of ring closure [Fig. 2 ▸(*b*)]. Though the resolution of these states (∼5 Å resolution) was improved compared with the initial MAC structure, local resolution estimates across the map suggested that the MAC was a highly flexible complex. By focusing image alignment on subsections of the complex, a series of smaller local reconstructions around the ring were calculated and stitched together to generate a composite map with higher resolution information. Atomic models derived from crystal structures of complement proteins, together with homology models for those whose structures were unavailable, served as a starting point to interpret higher-resolution features and provide a molecular basis for MAC assembly [Fig. 2 ▸(*c*)].

The MAC pore is formed by the polymerization of proteins containing a membrane-attack-complex/perforin (MACPF) fold. Despite a lack of sequence identity, MACPF domains are a highly conserved structural fold found across immune pores and bacterial effectors alike (Rossjohn *et al.*, 1997 ▸; Hadders *et al.*, 2007 ▸; Rosado *et al.*, 2007 ▸). The domain comprises a central ‘L-shaped’ β-sheet flanked by two α-helical regions. During pore formation, these helices undergo a dramatic secondary-structure rearrangement and transition into membrane-spanning β-hairpins (Dudkina *et al.*, 2016 ▸; Serna *et al.*, 2016 ▸). These higher-resolution MAC structures showed that electrostatic complementarity of MACPF interaction interfaces drives the directionality of the sequential assembly of complement proteins (Menny *et al.*, 2018 ▸). Genetic variants in C9 associated with risk of age-related macular degeneration map directly to the polymerization interface and suggest a role of polymerization in complement-mediated disease (McMahon *et al.*, 2021 ▸).

Polymerization of complement proteins is a highly regulated process. By comparing pore conformations of complement proteins (Menny *et al.*, 2018 ▸) with soluble states solved by X-ray crystallography (Spicer *et al.*, 2018 ▸; Hadders *et al.*, 2012 ▸; Lovelace *et al.*, 2011 ▸), it became obvious that the polymerization interface of each MACPF domain is only accessible once integrated into the membrane-bound assembly. This prevents off-target complexes from forming and avoids the depletion of complement proteins in serum. C8γ is a regulatory protein embedded within the heterotrimeric C8 complex. The crystal structure of C8 revealed that C8γ packs against the MACPF domain of C8α and blocks access to C9 (Lovelace *et al.*, 2011 ▸). Upon incorporation into the MAC, C8γ rotates from its position in the soluble conformation and frees the polymerization interface of C8α to recruit C9 (Serna *et al.*, 2016 ▸). Once bound to the nascent MAC, the helix-to-hairpin transition of C9 pore-forming residues removes a steric block to facilitate polymerization of C9 (Spicer *et al.*, 2018 ▸).

In addition to influencing the accessibility of protein interaction interfaces, the sequential assembly of complement proteins also affects the local lipid environment. MAC cryo-EM structures showed that the pore-forming β-hairpins of C6, C7 and C8β were shorter than those of C8α and C9, and that these residues did not span the full width of a standard lipid bilayer (Serna *et al.*, 2016 ▸; Menny *et al.*, 2018 ▸). In the MAC, C9 transmembrane residues form a contiguous hydrophobic belt that interacts with lipid tails. By contrast, the shorter hairpins of C6, C7 and C8 create a nonplanar positively charged patch that binds lipid headgroups and lowers the energy required to bend the lipid bilayer [Fig. 2 ▸(*d*)]. These data provided the first evidence that complement activation alters membrane properties and raised new questions about how those changes influence recruitment of membrane-bound regulators during an immune response.

## 3. MAC regulation

Complement regulators play a crucial role in controlling membrane damage and preventing human disease. Once activated on a surface, MAC assembly proceeds like an irreversible cascade of dominoes, creating pores in both prokaryotic and eukaryotic cells. During an innate immune response, cells are protected from the MAC through two main routes: one that captures and clears soluble complement activation byproducts and a second that stops the cascade of dominoes on the surface of host cells. MAC byproducts are important biomarkers of complement activation in human disease (Schoettler *et al.*, 2023 ▸; Chen *et al.*, 2025 ▸), and an off-target MAC can attack nearby macrophages with knock-on consequences for inflammation (Suresh *et al.*, 2016 ▸). Furthermore, low-level MAC activation on human cells contributes to chronic inflammatory disease (Jimenez-Duran *et al.*, 2022 ▸), while a MAC activated on red blood cells lacking complement regulators is a major driver of haemolytic anaemia (Nevo *et al.*, 2013 ▸).

Cryo-EM has had a profound impact on our understanding of how complement regulators control MAC activity. A low-resolution (24 Å) structure of the soluble MAC activation byproduct (sMAC or sC5b9) showed that the complex resembled an arc-like MAC precursor (Hadders *et al.*, 2012 ▸); however, it remained unclear how chaperones prevented polymerization of complement proteins. Combining advances in mass spectrometry and state-of-the-art cryo-EM, new sMAC structures revealed the true heterogeneity of the complex in blood (Menny *et al.*, 2021 ▸). Analysing samples purified from native material, these methods showed that sMAC could have multiple copies of each chaperone and varying numbers of C9. Crosslinking mass spectrometry identified complement protein residues near chaperones and indicated areas of the map on which to focus further classification and refinement. The resolution of the resulting map (3.3 Å) was much improved. These data then showed how the chaperone clusterin binds an arc of complement proteins through electrostatic interactions and caps the polymerizing face of C9.

**Figure 3:**
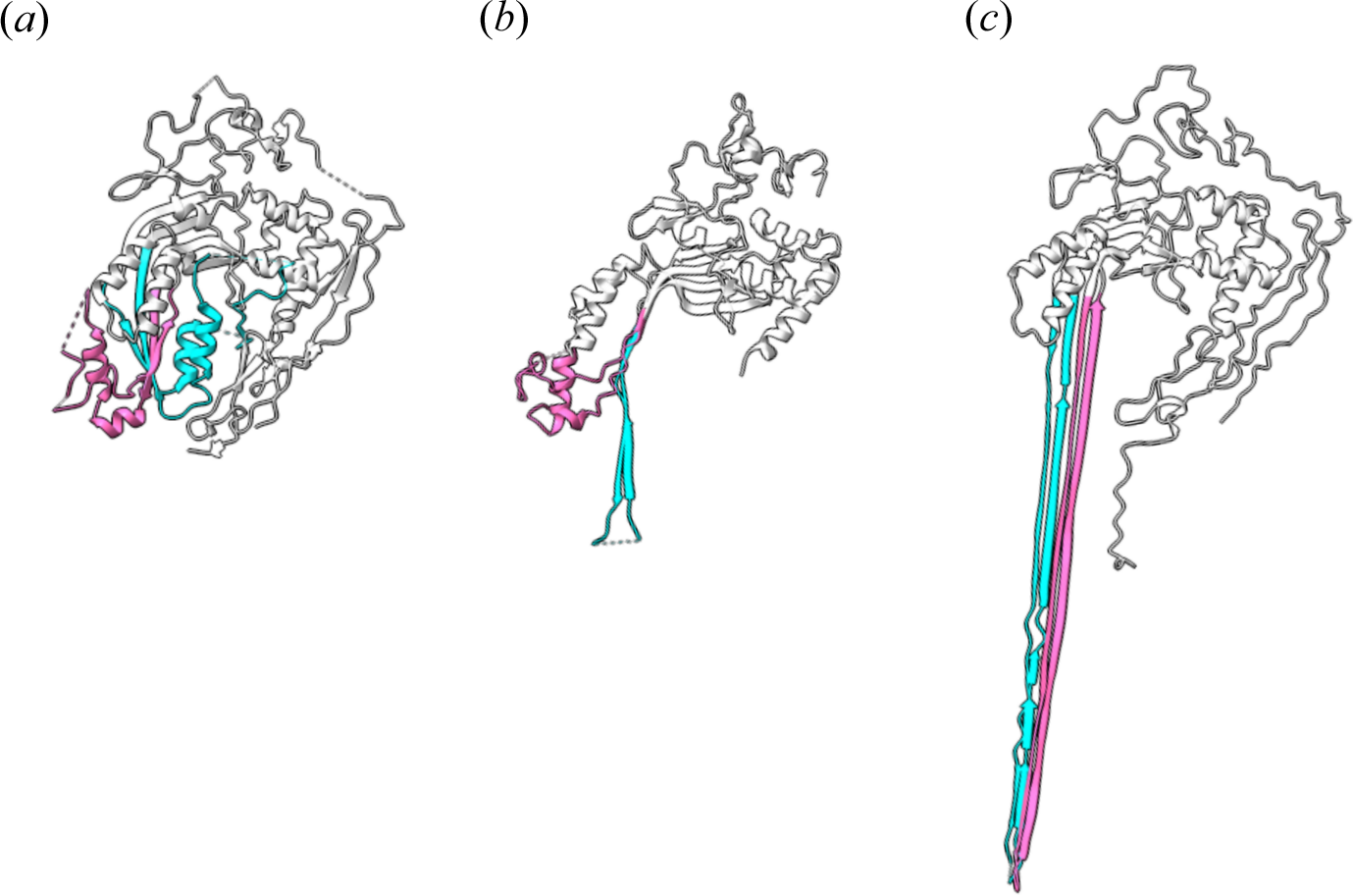
Atomic models for C9 MACPF domain conformations present in the soluble crystal structure [PDB entry 6cxo, (*a*); Spicer *et al.*, 2018 ▸], the inhibited sMAC [PDB entry 7nyd, (*b*); Menny *et al.*, 2021 ▸] and the transmembrane pore [PDB entry 6h04, (*c*)]. Residues that undergo the helix-to-hairpin transition are coloured (TMH1, cyan; TMH2, pink).

By blocking MAC assembly, the sMAC structure captured a previously unseen C9 conformation. During pore formation, the transmembrane residues of C9 transition from two helical bundles to two β-hairpins that insert into the lipid bilayer (Fig. 3 ▸). While some C9 molecules had completed this transition in sMAC, the final clusterin-capped C9 remained partially unfurled (Menny *et al.*, 2021 ▸). Locking the helical conformation of these residues could also inhibit C9 polymerization (Spicer *et al.*, 2018 ▸). Together these data led to the hypothesis that pore propagation depends on the complete helix-to-hairpin transition for both sets of β-hairpins.

Regulation of MAC assembly on cells is controlled by the plasma membrane receptor CD59 (Davies *et al.*, 1989 ▸). This widely expressed GPI-anchored protein is the body’s last line of defence against the complement terminal pathway and is the only membrane-bound MAC regulator. Genetic deficiency of GPI-anchored proteins, including CD59, results in devastating human disease and lysis of red blood cells during immune activation (Nevo *et al.*, 2013 ▸). Despite its role in protecting cells from MAC-mediated damage, CD59 can be co-opted by cancer cells as an immune evasion mechanism, contributing to immunotherapy resistance (Fishelson *et al.*, 2003 ▸; Shao *et al.*, 2022 ▸). Therefore, understanding how CD59 controls MAC activity is essential for understanding the role of complement in both health and disease.

**Figure 4:**
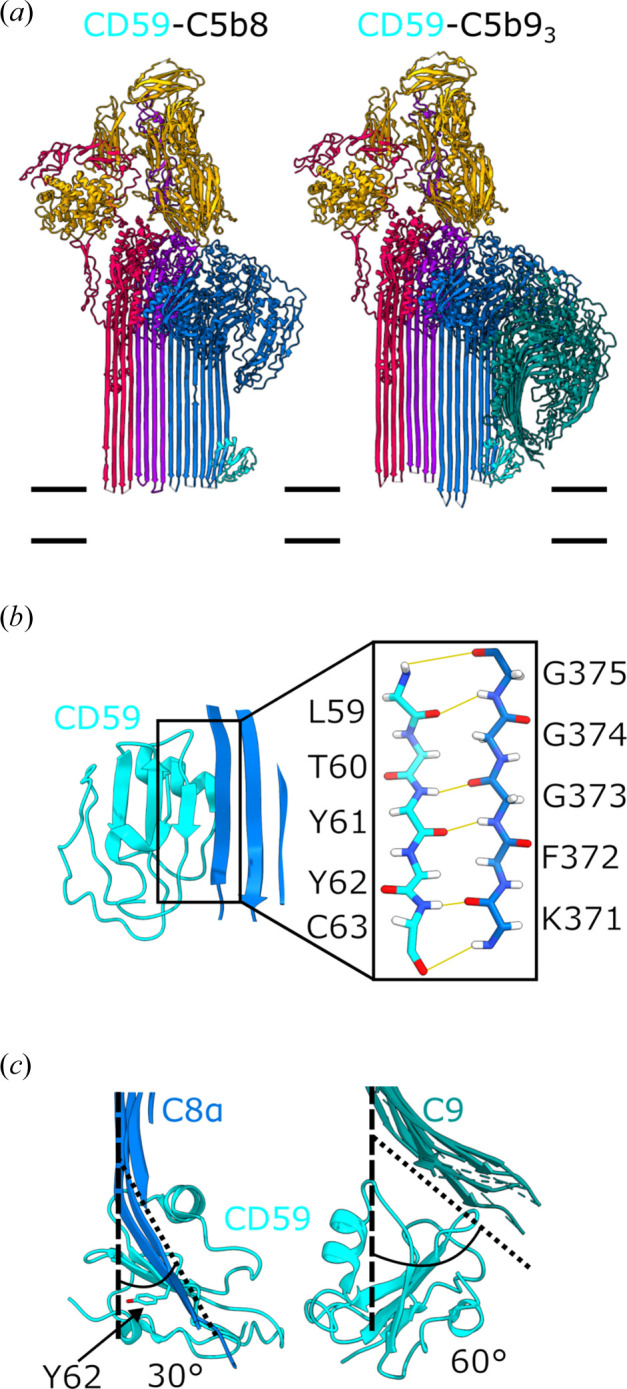
(*a*) Atomic models derived from the two CD59-inhibited MAC complexes: CD59-C5b8 and CD59-C5b9. Complement proteins coloured: C5b (orange), C6 (red), C7 (purple), C8 (blue) and C9 (turquoise); CD59 in cyan. The position of the membrane relative to the models is indicated as double black lines. (*b*) Ribbon diagram showing the intermolecular anti-parallel β-sheet formed between CD59 (cyan) and C8α (blue). The inset highlights the hydrogen-bonding network of the backbone interactions within the β-sheet. [(*c*), left panel] CD59 (cyan) deflects the β-hairpins of C8α (blue) 30° from their trajectory towards the membrane. CD59:Y62, which is important for both MAC inhibition and bacterial pore formation, is highlighted in sticks representation. [(*c*), right panel] CD59 sterically blocks the membrane trajectory of two C9 molecules’ β-hairpins (C9, turquoise), deflecting them 60° from their MAC conformation.

Using cryo-EM to visualize complexes in a membrane environment provided a unique opportunity to understand how CD59 inhibits a MAC (Supplementary Movie 1). Without its GPI anchor, soluble CD59 is a poor MAC inhibitor, suggesting that membrane localization plays an important role in binding complement proteins. Antibody mapping experiments showed that CD59 can interact with two MAC components, C8 and C9 (Huang *et al.*, 2006 ▸; Lockert *et al.*, 1995 ▸); however, it was unclear if binding both proteins at the membrane was necessary to block the pore. To capture inhibited MAC states, lipid nanodiscs were decorated with ectodomains of CD59 engineered with a synthetic GPI-anchor (Shah *et al.*, 2020 ▸; Couves *et al.*, 2023 ▸). CD59-decorated nanodiscs then served as a trap to catch the MAC as it assembled. Fragile complexes were stabilized with a mild crosslinker during density centrifugation, a sample preparation method called GraFix shown to work well for other large macromolecular assemblies (Stark, 2010 ▸). These methods trapped two discrete inhibited MAC complexes: one comprising a single copy of the MAC precursor C5b8 (solved at 3.0 Å resolution) and a second that also included C9 (C5b9, solved at 3.3 Å resolution) [Fig. 4 ▸(*a*)]. These structures showed that CD59 binds C8 and C9 simultaneously, suggesting C8-binding is prerequisite for blocking C9 membrane perforation and polymerization.

At the membrane, CD59 catches the helix-to-hairpin transition of the C8α MACPF domain (Couves *et al.*, 2023 ▸). While the first helical bundle of C8α unfurls to anchor the complex to the outer leaflet of the lipid bilayer, the membrane trajectory of the second set of residues is altered by CD59 [Fig. 4 ▸(*c*)]. The interaction is stabilized by a salt bridge across the two proteins at the start of the hairpin. Meanwhile, a highly conserved aromatic side chain of CD59 bends a glycine-rich region of C8α and changes the angle of the hairpin. The central β-sheet of CD59 templates the newly formed C8α hairpin through an extensive hydrogen-bonding network comprised largely of backbone atoms. The resulting intermolecular β-sheet forms a stable and irreversible complex on the membrane [Fig. 4 ▸(*b*)]. This complex provided important molecular insights into how human cells are protected from MAC damage. Using this structure, inhibited complexes from other species could be modelled, revealing that co-evolved interface residues underlie species-specific complement regulation. In addition to binding C8α, CD59 also binds bacterial pore-forming proteins that co-opt the complement regulator during infection. Through the same C8α-binding interface, CD59 recognizes a β-hairpin that extends from the membrane-binding domain of the bacterial protein (Johnson *et al.*, 2013 ▸). Despite a shared interaction interface, there is limited sequence similarity between CD59’s two binding partners. These structures underpinned the development of novel macrocyclic peptide-based complement regulators that control CD59 activity through a conserved secondary structural motif (Bickel *et al.*, 2025 ▸).

By binding the C8α β-hairpin, CD59 is primed to block membrane insertion and polymerization of C9. The cryo-EM structure of the inhibited C5b8 complex showed that although CD59 deflects the pore-forming β-hairpins of C8α, the polymerization interface of the MACPF domain remained exposed (Couves *et al.*, 2023 ▸). Consequently, C9-binding triggers conformational changes in its MACPF domain that unfurl both sets of transmembrane β-hairpins. In the inhibited complex, CD59 is located directly below the path of these cascading β-hairpins and prevents them from reaching the lipid bilayer [Figs. 4 ▸(*a*) and 4 ▸(*d*)]. With both sets of C9 hairpins released, the polymerization interface remains exposed, ready for the next C9 molecule to join the assembly. How then does CD59 stop the dominoes from falling? The long β-hairpins of newly bound complement proteins are templated by the growing β-barrel of the assembly precursor. In the inhibited complex, the altered trajectory of the C9 hairpins disrupts the alignment of subsequent C9 molecules, so that eventually only one of the two hairpins is released. In this way, CD59 indirectly stops C9 polymerization and traps a conformation of C9 observed in the capped sMAC structure [Fig. 3 ▸(*b*)], and suggests a general mechanism for controlling MAC activity (Menny *et al.*, 2021 ▸).

In addition to protein–protein interactions, cryo-EM structures of the MAC inhibited on membranes provided direct evidence for membrane distortions caused by immune activation. Earlier MAC structures showed that complement proteins could interact with lipids in two ways, depending on the length and charged properties of their β-hairpins (Menny *et al.*, 2018 ▸). The structure of the inhibited MAC precursor complex showed how these shorter β-hairpins locally thin the bilayer before the pore is formed (Couves *et al.*, 2023 ▸). Molecular-dynamics simulations supported these structural observations and confirmed that although these residues anchor into the outer leaflet, the membrane remains impermeable to water (Couves *et al.*, 2023 ▸). Additional atomistic simulations of a GPI-anchored CD59 revealed that lipid head groups form extensive hydrogen bonds with residues within the complement binding interface (Voisin *et al.*, 2023 ▸). Therefore, local changes in the lipid environment are likely required to free the interface and allow CD59 to block C8α.

These mechanistic insights into MAC regulation were only possible because of advances in structural biology methods. In addition to algorithms that address discrete heterogeneity, CD59-inhibited complexes required new computational tools that could handle continuous flexibility of the complex (Zhong *et al.*, 2021 ▸; Punjani & Fleet, 2021 ▸). Although these approaches significantly improved the resolution of the maps, they were still well below the threshold for *de novo* model building. *AlphaFold* is a machine-learning-based structural-prediction tool that has transformed model building in moderate-resolution cryo-EM maps (Jumper *et al.*, 2021 ▸). Leveraging the power of *AlphaFold*, these inhibited MAC structures provided a robust molecular model for complement protein interactions. Finally, innovative lipid systems that incorporated synthetic GPI-anchored proteins provided insight into how the MAC is controlled on model membranes. Looking ahead, emerging technologies in structural cell biology will open new avenues to explore complement regulation in a more physiologically relevant context.

## 4. Future perspectives

A decade later, advances in machine learning and artificial intelligence are driving the next frontier of the cryo-EM revolution. Deep-learning approaches from the field of computer vision are transforming every aspect of structural biology. Denoising convolutional neural networks are expanding the capability of cryo-EM image analysis to include smaller complexes and have led to improved reconstructions from images with low signal-to-noise ratios (Kimanius *et al.*, 2024 ▸). In addition, neural field networks are being used to improve the accuracy of cryo-EM reconstructions in real-space refinements (Huang *et al.*, 2024 ▸). While these advances are improving the resolution of homogeneous structures, new tools are emerging in image heterogeneity analysis (Schwab *et al.*, 2024 ▸). Together, these approaches offer the possibility for visualizing transient and rare conformations of complement protein complexes using single-particle analyses.

At the same time, the resolution of cellular imaging by cryo-electron tomography is close on the heels of single-particle cryo-EM. Many of the same denoising and classification methods developed for 2D image analyses are now being applied to 3D tomograms. Deep-learning-based methods are being deployed to pick 3D particles in crowded and noisy cryo-tomograms of cells (Liu *et al.*, 2024 ▸). Convolutional neural networks and U-Net architectures are automating segmentation of these data, shortening the time from data collection to mechanistic discovery (de Teresa-Trueba *et al.*, 2023 ▸). As we transition into the next frontier of cryo-EM, these tools will allow us to study the MAC in a cellular context and discover new biology that will drive the next generation of complement therapeutics.

## Supplementary Material

Supplementary Movie 1: MAC assembly and regulation. DOI: 10.1107/S2052252526002927/eh5025sup1.mp4
